# Bis{1-[(1*H*-benzimidazol-1-yl)methyl-κ*N* ^3^]-1*H*-1,2,3,4-tetra­zole}silver(I) nitrate

**DOI:** 10.1107/S1600536810006653

**Published:** 2010-02-27

**Authors:** Huai-xia Yang, Xia Wang, Ya-nan Ding, Xiang-ru Meng

**Affiliations:** a Pharmacy College, Henan University of Traditional Chinese Medicine, Zhengzhou 450008, People’s Republic of China; b Department of Chemistry, Zhengzhou University, Zhengzhou 450052, People’s Republic of China

## Abstract

In the title salt, [Ag(C_9_H_8_N_6_)_2_]NO_3_, the central Ag^I^ atom is linearly coordinated by the N atoms [171.97 (8)°]  from two 1-[(benzimidazol-1-yl)meth­yl]-1*H*-1,2,3,4-tetra­zole ligands. The benzimidazole rings in adjacent mol­ecules are parallel with an average inter­planar distance of 3.461 Å; adjacent mol­ecules are linked through N—H⋯O hydrogen bonds into a linear chain along the *b*-axis direction.

## Related literature

For similar compounds, see: Bronisz (2004 ▶); Meng *et al.* (2009 ▶, 2004 ▶); Huang *et al.* (2006 ▶). 
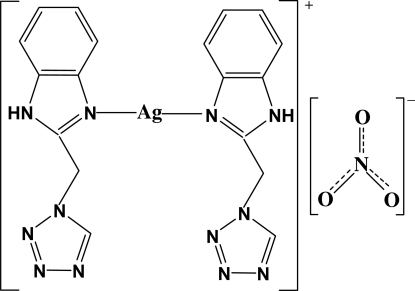


## Experimental

### 

#### Crystal data

[Ag(C_9_H_8_N_6_)_2_]NO_3_
*M* *_r_* = 570.31
Monoclinic, 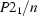

*a* = 11.125 (2) Å
*b* = 9.3276 (19) Å
*c* = 20.189 (4) Å
β = 94.80 (3)°
*V* = 2087.6 (7) Å^3^
*Z* = 4
Mo *K*α radiation
μ = 1.02 mm^−1^
*T* = 293 K
0.22 × 0.18 × 0.17 mm

#### Data collection

Rigaku Saturn diffractometer
Absorption correction: multi-scan (*CrystalClear*; Rigaku/MSC, 2006 ▶) *T*_min_ = 0.807, *T*_max_ = 0.846
25286 measured reflections
4974 independent reflections
4605 reflections with *I* > 2σ(*I*)
*R*_int_ = 0.033

#### Refinement

*R*[*F* ^2^ > 2σ(*F* ^2^)] = 0.041
*wR*(*F* ^2^) = 0.106
*S* = 1.03
4974 reflections
316 parameters
H-atom parameters constrained
Δρ_max_ = 0.58 e Å^−3^
Δρ_min_ = −0.46 e Å^−3^

### 

Data collection: *CrystalClear* (Rigaku/MSC, 2006 ▶); cell refinement: *CrystalClear*; data reduction: *CrystalClear*; program(s) used to solve structure: *SHELXS97* (Sheldrick, 2008 ▶); program(s) used to refine structure: *SHELXL97* (Sheldrick, 2008 ▶); molecular graphics: *SHELXTL* (Sheldrick, 2008 ▶); software used to prepare material for publication: *SHELXTL*.

## Supplementary Material

Crystal structure: contains datablocks global, I. DOI: 10.1107/S1600536810006653/ng2735sup1.cif

Structure factors: contains datablocks I. DOI: 10.1107/S1600536810006653/ng2735Isup2.hkl

Additional supplementary materials:  crystallographic information; 3D view; checkCIF report

## Figures and Tables

**Table 1:** Hydrogen-bond geometry (Å, °)

*D*—H⋯*A*	*D*—H	H⋯*A*	*D*⋯*A*	*D*—H⋯*A*
N8—H8*C*⋯O3	0.86	2.32	3.105 (5)	153
N8—H8*C*⋯O2	0.86	2.40	3.176 (4)	151
N2—H2*B*⋯O1^i^	0.86	2.06	2.881 (4)	159

Symmetry code: (i) 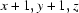.

**Fig. 1.**
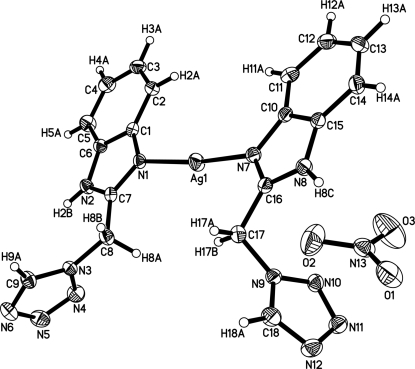
View of the title complex, showing the labeling of the 30% probability ellipsolids.

## supplementary crystallographic information

### Comment

In coordination and metallosupramolecular chemistry, there are many symmetrical tetrazole and benzimidazole ligands, which have been widely used as the classical ligands (Bronisz 2004; Meng *et al.* 2004). However, studies on unsymmetrical ligands concerning tetrazole and benzimidazole are rather insufficient (Meng *et al.* 2009; Huang *et al.* 2006). We are engaged in the synthesis of unsymmetrical N-heterocyclic ligands and synthesized compound 1-((benzimidazole-1-yl)methyl)-1-H-1,2,3,4-tetrazole. In this work, we selected this compound as ligand and generate a new complex [Ag(C_9_H_8_N_6_)_2_](NO_3_), (I), which is reported here. In complex (I) each Ag^I^ ion is two-coordinated by two N atom from two benzimidazole units and the nitrate anion does not coordinate to Ag^I^ ion (Fig. 1). Benzimidazole rings between the adjacent molecules are stacked in a face-to-face orientation with the distance of 3.461 Å. [Ag(C_9_H_8_N_6_)_2_](NO_3_) units are linked through these π–π interactions and various kinds of hydrogen bonds such as N—H···O, C—H···O, and C—H···N hydrogen bonds resulting in a three-dimensional packing structure in solid state as shown in Fig. 2.

### Experimental

The ligand 1-((benzimidazole-1-yl)methyl)-1*H*-1,2,3,4-tetrazole (0.1 mmol, 0.020 g) in methanol (6 ml) was added dropwise to a solution of AgNO_3_ (0.05 mmol, 0.008 g) in H_2_O (3 ml). The resulting solution was allowed to stand at room temperature in the dark. After four weeks good quality colorless crystals were obtained from the filtrate and dried in air.

### Crystal data

**Table:**

[Ag(C_9_H_8_N_6_)_2_]NO_3_	*F*(000) = 1144
*M**_r_* = 570.31	*D*_x_ = 1.815 Mg m^−^^3^
Monoclinic, *P*2_1_/*n*	Mo *K*α radiation, λ = 0.71073 Å
*a* = 11.125 (2) Å	Cell parameters from 6007 reflections
*b* = 9.3276 (19) Å	θ = 2.0–27.9°
*c* = 20.189 (4) Å	µ = 1.02 mm^−^^1^
β = 94.80 (3)°	*T* = 293 K
*V* = 2087.6 (7) Å^3^	Prism, colorless
*Z* = 4	0.22 × 0.18 × 0.17 mm

### Data collection

**Table:**

Rigaku Saturn diffractometer	4974 independent reflections
Radiation source: fine-focus sealed tube	4605 reflections with *I* > 2σ(*I*)
graphite	*R*_int_ = 0.033
Detector resolution: 28.5714 pixels mm^-1^	θ_max_ = 27.9°, θ_min_ = 2.4°
ω scans	*h* = −14→14
Absorption correction: multi-scan (*CrystalClear*; Rigaku/MSC, 2006)	*k* = −12→12
*T*_min_ = 0.807, *T*_max_ = 0.846	*l* = −25→26
25286 measured reflections	

### Refinement

**Table:**

Refinement on *F*^2^	Primary atom site location: structure-invariant direct methods
Least-squares matrix: full	Secondary atom site location: difference Fourier map
*R*[*F*^2^ > 2σ(*F*^2^)] = 0.041	Hydrogen site location: inferred from neighbouring sites
*wR*(*F*^2^) = 0.106	H-atom parameters constrained
*S* = 1.03	*w* = 1/[σ^2^(*F*_o_^2^) + (0.056*P*)^2^ + 1.4891*P*] where *P* = (*F*_o_^2^ + 2*F*_c_^2^)/3
4974 reflections	(Δ/σ)_max_ < 0.001
316 parameters	Δρ_max_ = 0.58 e Å^−^^3^
0 restraints	Δρ_min_ = −0.46 e Å^−^^3^

### Special details

**Table:**

Geometry. All esds (except the esd in the dihedral angle between two l.s. planes) are estimated using the full covariance matrix. The cell esds are taken into account individually in the estimation of esds in distances, angles and torsion angles; correlations between esds in cell parameters are only used when they are defined by crystal symmetry. An approximate (isotropic) treatment of cell esds is used for estimating esds involving l.s. planes.
Refinement. Refinement of *F*^2^ against ALL reflections. The weighted *R*-factor wR and goodness of fit *S* are based on *F*^2^, conventional *R*-factors *R* are based on *F*, with *F* set to zero for negative *F*^2^. The threshold expression of *F*^2^ > σ(*F*^2^) is used only for calculating *R*-factors(gt) etc. and is not relevant to the choice of reflections for refinement. *R*-factors based on *F*^2^ are statistically about twice as large as those based on *F*, and *R*- factors based on ALL data will be even larger.

### Fractional atomic coordinates and isotropic or equivalent isotropic displacement parameters (Å^2^)

**Table:**

	*x*	*y*	*z*	*U*_iso_*/*U*_eq_	
Ag1	0.43599 (2)	0.61837 (3)	0.079718 (11)	0.04196 (10)	
N1	0.59520 (18)	0.7426 (2)	0.08358 (11)	0.0287 (4)	
N2	0.7519 (2)	0.8683 (2)	0.12658 (12)	0.0321 (5)	
H2B	0.8090	0.8972	0.1548	0.039*	
N3	0.7700 (2)	0.6972 (2)	0.24677 (11)	0.0300 (5)	
N4	0.7703 (2)	0.8136 (3)	0.28571 (13)	0.0447 (6)	
N5	0.8649 (3)	0.8034 (3)	0.32741 (13)	0.0505 (7)	
N6	0.9263 (2)	0.6814 (3)	0.31659 (13)	0.0452 (6)	
N7	0.2869 (2)	0.4769 (2)	0.06530 (11)	0.0320 (5)	
N8	0.1619 (2)	0.2970 (2)	0.07878 (11)	0.0330 (5)	
H8C	0.1284	0.2262	0.0973	0.040*	
N9	0.2313 (2)	0.3251 (2)	0.22291 (11)	0.0302 (5)	
N10	0.1337 (2)	0.4074 (3)	0.22742 (12)	0.0360 (5)	
N11	0.0800 (2)	0.3607 (3)	0.27719 (13)	0.0415 (6)	
N12	0.1414 (3)	0.2475 (3)	0.30616 (13)	0.0452 (6)	
N13	0.0308 (2)	0.0190 (3)	0.17241 (12)	0.0433 (6)	
C1	0.6263 (2)	0.8422 (3)	0.03637 (13)	0.0294 (5)	
C2	0.5768 (3)	0.8669 (3)	−0.02817 (14)	0.0350 (6)	
H2A	0.5120	0.8133	−0.0467	0.042*	
C3	0.6278 (3)	0.9742 (3)	−0.06341 (14)	0.0414 (7)	
H3A	0.5972	0.9924	−0.1069	0.050*	
C4	0.7241 (3)	1.0564 (4)	−0.03570 (16)	0.0449 (7)	
H4A	0.7550	1.1291	−0.0609	0.054*	
C5	0.7746 (3)	1.0324 (3)	0.02809 (16)	0.0428 (7)	
H5A	0.8390	1.0867	0.0466	0.051*	
C6	0.7239 (2)	0.9228 (3)	0.06337 (13)	0.0306 (5)	
C7	0.6732 (2)	0.7616 (3)	0.13568 (13)	0.0286 (5)	
C8	0.6710 (3)	0.6705 (3)	0.19633 (14)	0.0362 (6)	
H8A	0.5954	0.6864	0.2158	0.043*	
H8B	0.6734	0.5705	0.1832	0.043*	
C9	0.8655 (3)	0.6184 (3)	0.26680 (15)	0.0373 (6)	
H9A	0.8859	0.5315	0.2482	0.045*	
C10	0.2093 (2)	0.4638 (3)	0.00770 (13)	0.0302 (5)	
C11	0.2002 (3)	0.5461 (3)	−0.04975 (14)	0.0393 (6)	
H11A	0.2533	0.6211	−0.0556	0.047*	
C12	0.1095 (3)	0.5122 (4)	−0.09771 (15)	0.0445 (7)	
H12A	0.0993	0.5677	−0.1360	0.053*	
C13	0.0325 (3)	0.3965 (4)	−0.09021 (16)	0.0482 (8)	
H13A	−0.0260	0.3748	−0.1243	0.058*	
C14	0.0409 (3)	0.3135 (4)	−0.03344 (15)	0.0409 (7)	
H14A	−0.0109	0.2367	−0.0284	0.049*	
C15	0.1305 (2)	0.3501 (3)	0.01585 (14)	0.0309 (5)	
C16	0.2541 (2)	0.3759 (3)	0.10575 (14)	0.0311 (6)	
C17	0.3165 (3)	0.3487 (4)	0.17282 (14)	0.0386 (6)	
H17A	0.3679	0.2651	0.1707	0.046*	
H17B	0.3674	0.4301	0.1858	0.046*	
C18	0.2339 (3)	0.2295 (3)	0.27144 (15)	0.0413 (7)	
H18A	0.2930	0.1598	0.2796	0.050*	
O1	−0.0183 (3)	−0.0797 (3)	0.19941 (14)	0.0774 (10)	
O2	0.1406 (3)	0.0210 (4)	0.16912 (17)	0.0827 (9)	
O3	−0.0271 (4)	0.1221 (4)	0.1498 (2)	0.1045 (14)	

### Atomic displacement parameters (Å^2^)

**Table:**

	*U*^11^	*U*^22^	*U*^33^	*U*^12^	*U*^13^	*U*^23^
Ag1	0.03417 (14)	0.04656 (16)	0.04425 (16)	−0.01549 (9)	−0.00206 (10)	0.00349 (10)
N1	0.0227 (10)	0.0308 (11)	0.0324 (11)	−0.0037 (8)	0.0008 (8)	0.0011 (9)
N2	0.0289 (11)	0.0329 (11)	0.0336 (12)	−0.0057 (9)	−0.0038 (9)	0.0027 (9)
N3	0.0313 (11)	0.0287 (11)	0.0296 (11)	0.0018 (9)	−0.0009 (9)	0.0013 (9)
N4	0.0503 (16)	0.0381 (13)	0.0455 (15)	0.0081 (12)	0.0027 (12)	−0.0096 (11)
N5	0.0604 (18)	0.0486 (16)	0.0410 (15)	−0.0031 (13)	−0.0035 (13)	−0.0090 (12)
N6	0.0443 (15)	0.0448 (14)	0.0438 (15)	−0.0016 (12)	−0.0119 (11)	0.0039 (12)
N7	0.0299 (11)	0.0349 (12)	0.0313 (12)	−0.0058 (9)	0.0034 (9)	−0.0035 (9)
N8	0.0311 (11)	0.0342 (12)	0.0337 (12)	−0.0066 (9)	0.0027 (9)	0.0043 (9)
N9	0.0305 (11)	0.0312 (11)	0.0286 (11)	0.0035 (9)	0.0022 (9)	0.0027 (9)
N10	0.0312 (12)	0.0371 (12)	0.0397 (13)	0.0050 (9)	0.0022 (10)	0.0052 (10)
N11	0.0413 (14)	0.0438 (14)	0.0401 (14)	0.0019 (11)	0.0078 (11)	−0.0003 (11)
N12	0.0519 (16)	0.0451 (15)	0.0398 (14)	0.0022 (12)	0.0106 (12)	0.0096 (11)
N13	0.0479 (16)	0.0464 (15)	0.0344 (13)	−0.0141 (12)	−0.0037 (11)	0.0009 (11)
C1	0.0243 (12)	0.0310 (12)	0.0334 (14)	0.0011 (10)	0.0053 (10)	−0.0006 (10)
C2	0.0333 (14)	0.0418 (15)	0.0295 (14)	0.0011 (11)	−0.0003 (11)	−0.0023 (11)
C3	0.0475 (17)	0.0470 (17)	0.0297 (14)	0.0088 (14)	0.0039 (12)	0.0040 (12)
C4	0.0507 (19)	0.0415 (16)	0.0437 (17)	−0.0041 (14)	0.0108 (14)	0.0093 (14)
C5	0.0428 (17)	0.0389 (15)	0.0462 (17)	−0.0119 (13)	0.0007 (13)	0.0035 (13)
C6	0.0296 (13)	0.0317 (13)	0.0305 (13)	−0.0008 (10)	0.0023 (10)	0.0009 (11)
C7	0.0226 (11)	0.0294 (12)	0.0336 (13)	−0.0001 (9)	0.0010 (10)	0.0002 (10)
C8	0.0310 (14)	0.0381 (14)	0.0384 (15)	−0.0054 (11)	−0.0034 (11)	0.0081 (12)
C9	0.0366 (15)	0.0328 (14)	0.0413 (16)	0.0049 (11)	−0.0046 (12)	−0.0004 (11)
C10	0.0268 (12)	0.0335 (13)	0.0310 (13)	−0.0006 (10)	0.0058 (10)	−0.0049 (11)
C11	0.0459 (17)	0.0396 (15)	0.0342 (15)	0.0011 (13)	0.0129 (12)	0.0037 (12)
C12	0.0477 (18)	0.0555 (19)	0.0308 (15)	0.0114 (15)	0.0052 (13)	0.0029 (13)
C13	0.0405 (17)	0.069 (2)	0.0337 (16)	0.0098 (15)	−0.0039 (13)	−0.0109 (15)
C14	0.0309 (14)	0.0471 (17)	0.0442 (17)	−0.0037 (12)	0.0003 (12)	−0.0071 (14)
C15	0.0266 (13)	0.0347 (13)	0.0315 (14)	−0.0007 (10)	0.0044 (10)	−0.0033 (11)
C16	0.0266 (13)	0.0361 (14)	0.0310 (14)	−0.0037 (10)	0.0049 (10)	−0.0023 (11)
C17	0.0296 (14)	0.0495 (17)	0.0365 (15)	−0.0010 (12)	0.0016 (11)	0.0034 (13)
C18	0.0450 (17)	0.0364 (15)	0.0422 (17)	0.0068 (12)	0.0012 (13)	0.0095 (13)
O1	0.0706 (18)	0.092 (2)	0.0654 (17)	−0.0480 (17)	−0.0162 (14)	0.0345 (16)
O2	0.0586 (18)	0.090 (2)	0.103 (2)	−0.0185 (16)	0.0260 (17)	−0.0062 (19)
O3	0.112 (3)	0.073 (2)	0.120 (3)	0.0034 (19)	−0.046 (3)	0.024 (2)

### Geometric parameters (Å, °)

**Table:**

Ag1—N1	2.112 (2)	C1—C6	1.393 (4)
Ag1—N7	2.121 (2)	C2—C3	1.377 (4)
N1—C7	1.318 (3)	C2—H2A	0.9300
N1—C1	1.395 (3)	C3—C4	1.396 (5)
N2—C7	1.348 (3)	C3—H3A	0.9300
N2—C6	1.385 (4)	C4—C5	1.379 (4)
N2—H2B	0.8600	C4—H4A	0.9300
N3—C9	1.327 (4)	C5—C6	1.392 (4)
N3—N4	1.340 (3)	C5—H5A	0.9300
N3—C8	1.457 (3)	C7—C8	1.492 (4)
N4—N5	1.295 (4)	C8—H8A	0.9700
N5—N6	1.354 (4)	C8—H8B	0.9700
N6—C9	1.304 (4)	C9—H9A	0.9300
N7—C16	1.318 (3)	C10—C11	1.387 (4)
N7—C10	1.394 (3)	C10—C15	1.395 (4)
N8—C16	1.340 (3)	C11—C12	1.375 (4)
N8—C15	1.381 (3)	C11—H11A	0.9300
N8—H8C	0.8600	C12—C13	1.394 (5)
N9—C18	1.323 (3)	C12—H12A	0.9300
N9—N10	1.339 (3)	C13—C14	1.380 (5)
N9—C17	1.459 (4)	C13—H13A	0.9300
N10—N11	1.287 (4)	C14—C15	1.391 (4)
N11—N12	1.363 (4)	C14—H14A	0.9300
N12—C18	1.304 (4)	C16—C17	1.491 (4)
N13—O1	1.222 (3)	C17—H17A	0.9700
N13—O3	1.224 (4)	C17—H17B	0.9700
N13—O2	1.229 (4)	C18—H18A	0.9300
C1—C2	1.391 (4)		
			
N1—Ag1—N7	171.97 (8)	N2—C6—C1	105.7 (2)
C7—N1—C1	105.7 (2)	C5—C6—C1	122.1 (3)
C7—N1—Ag1	126.67 (18)	N1—C7—N2	112.5 (2)
C1—N1—Ag1	126.51 (17)	N1—C7—C8	121.5 (2)
C7—N2—C6	107.3 (2)	N2—C7—C8	126.0 (2)
C7—N2—H2B	126.3	N3—C8—C7	114.4 (2)
C6—N2—H2B	126.3	N3—C8—H8A	108.7
C9—N3—N4	107.9 (2)	C7—C8—H8A	108.7
C9—N3—C8	131.1 (2)	N3—C8—H8B	108.7
N4—N3—C8	120.9 (2)	C7—C8—H8B	108.7
N5—N4—N3	106.5 (2)	H8A—C8—H8B	107.6
N4—N5—N6	110.5 (2)	N6—C9—N3	109.6 (3)
C9—N6—N5	105.5 (2)	N6—C9—H9A	125.2
C16—N7—C10	105.7 (2)	N3—C9—H9A	125.2
C16—N7—Ag1	127.84 (19)	C11—C10—N7	130.2 (3)
C10—N7—Ag1	126.27 (18)	C11—C10—C15	121.1 (3)
C16—N8—C15	107.6 (2)	N7—C10—C15	108.6 (2)
C16—N8—H8C	126.2	C12—C11—C10	117.4 (3)
C15—N8—H8C	126.2	C12—C11—H11A	121.3
C18—N9—N10	107.7 (2)	C10—C11—H11A	121.3
C18—N9—C17	129.7 (2)	C11—C12—C13	121.5 (3)
N10—N9—C17	122.5 (2)	C11—C12—H12A	119.2
N11—N10—N9	106.9 (2)	C13—C12—H12A	119.2
N10—N11—N12	110.4 (2)	C14—C13—C12	121.7 (3)
C18—N12—N11	105.0 (2)	C14—C13—H13A	119.2
O1—N13—O3	121.2 (4)	C12—C13—H13A	119.2
O1—N13—O2	121.2 (3)	C13—C14—C15	116.8 (3)
O3—N13—O2	117.5 (3)	C13—C14—H14A	121.6
C2—C1—C6	120.8 (3)	C15—C14—H14A	121.6
C2—C1—N1	130.5 (2)	N8—C15—C14	133.0 (3)
C6—C1—N1	108.8 (2)	N8—C15—C10	105.5 (2)
C3—C2—C1	117.2 (3)	C14—C15—C10	121.5 (3)
C3—C2—H2A	121.4	N7—C16—N8	112.5 (2)
C1—C2—H2A	121.4	N7—C16—C17	123.6 (2)
C2—C3—C4	121.8 (3)	N8—C16—C17	123.9 (2)
C2—C3—H3A	119.1	N9—C17—C16	112.0 (2)
C4—C3—H3A	119.1	N9—C17—H17A	109.2
C5—C4—C3	121.6 (3)	C16—C17—H17A	109.2
C5—C4—H4A	119.2	N9—C17—H17B	109.2
C3—C4—H4A	119.2	C16—C17—H17B	109.2
C4—C5—C6	116.5 (3)	H17A—C17—H17B	107.9
C4—C5—H5A	121.7	N12—C18—N9	110.0 (3)
C6—C5—H5A	121.7	N12—C18—H18A	125.0
N2—C6—C5	132.2 (3)	N9—C18—H18A	125.0
			
N7—Ag1—N1—C7	−120.6 (6)	N4—N3—C8—C7	75.8 (3)
N7—Ag1—N1—C1	73.0 (7)	N1—C7—C8—N3	174.7 (2)
C9—N3—N4—N5	0.5 (3)	N2—C7—C8—N3	−4.5 (4)
C8—N3—N4—N5	176.4 (3)	N5—N6—C9—N3	0.2 (4)
N3—N4—N5—N6	−0.4 (4)	N4—N3—C9—N6	−0.4 (3)
N4—N5—N6—C9	0.1 (4)	C8—N3—C9—N6	−175.8 (3)
N1—Ag1—N7—C16	109.2 (6)	C16—N7—C10—C11	177.1 (3)
N1—Ag1—N7—C10	−64.6 (7)	Ag1—N7—C10—C11	−8.1 (4)
C18—N9—N10—N11	−0.3 (3)	C16—N7—C10—C15	−0.5 (3)
C17—N9—N10—N11	−178.4 (2)	Ag1—N7—C10—C15	174.33 (18)
N9—N10—N11—N12	0.1 (3)	N7—C10—C11—C12	−176.5 (3)
N10—N11—N12—C18	0.2 (4)	C15—C10—C11—C12	0.9 (4)
C7—N1—C1—C2	178.0 (3)	C10—C11—C12—C13	−2.4 (5)
Ag1—N1—C1—C2	−13.3 (4)	C11—C12—C13—C14	2.2 (5)
C7—N1—C1—C6	−1.4 (3)	C12—C13—C14—C15	−0.4 (5)
Ag1—N1—C1—C6	167.28 (18)	C16—N8—C15—C14	−178.1 (3)
C6—C1—C2—C3	−0.4 (4)	C16—N8—C15—C10	0.0 (3)
N1—C1—C2—C3	−179.8 (3)	C13—C14—C15—N8	176.8 (3)
C1—C2—C3—C4	−0.8 (4)	C13—C14—C15—C10	−1.1 (4)
C2—C3—C4—C5	1.2 (5)	C11—C10—C15—N8	−177.5 (2)
C3—C4—C5—C6	−0.4 (5)	N7—C10—C15—N8	0.3 (3)
C7—N2—C6—C5	180.0 (3)	C11—C10—C15—C14	0.9 (4)
C7—N2—C6—C1	−0.4 (3)	N7—C10—C15—C14	178.7 (3)
C4—C5—C6—N2	178.7 (3)	C10—N7—C16—N8	0.6 (3)
C4—C5—C6—C1	−0.8 (5)	Ag1—N7—C16—N8	−174.18 (18)
C2—C1—C6—N2	−178.4 (2)	C10—N7—C16—C17	178.0 (2)
N1—C1—C6—N2	1.1 (3)	Ag1—N7—C16—C17	3.3 (4)
C2—C1—C6—C5	1.3 (4)	C15—N8—C16—N7	−0.4 (3)
N1—C1—C6—C5	−179.3 (3)	C15—N8—C16—C17	−177.8 (3)
C1—N1—C7—N2	1.1 (3)	C18—N9—C17—C16	137.7 (3)
Ag1—N1—C7—N2	−167.49 (17)	N10—N9—C17—C16	−44.6 (4)
C1—N1—C7—C8	−178.2 (2)	N7—C16—C17—N9	136.4 (3)
Ag1—N1—C7—C8	13.2 (4)	N8—C16—C17—N9	−46.5 (4)
C6—N2—C7—N1	−0.5 (3)	N11—N12—C18—N9	−0.4 (4)
C6—N2—C7—C8	178.8 (3)	N10—N9—C18—N12	0.4 (3)
C9—N3—C8—C7	−109.3 (3)	C17—N9—C18—N12	178.4 (3)

### Hydrogen-bond geometry (Å, °)

**Table:**

*D*—H···*A*	*D*—H	H···*A*	*D*···*A*	*D*—H···*A*
N8—H8C···O3	0.86	2.32	3.105 (5)	153
N8—H8C···O2	0.86	2.40	3.176 (4)	151
N2—H2B···O1^i^	0.86	2.06	2.881 (4)	159
C5—H5A···O3^i^	0.93	2.48	3.271 (5)	143
C8—H8A···N11^ii^	0.97	2.55	3.388 (4)	144
C8—H8B···N4^iii^	0.97	2.54	3.406 (4)	148
C8—H8B···N5^iii^	0.97	2.53	3.476 (4)	164
C17—H17A···N6^iii^	0.97	2.41	3.250 (4)	145
C18—H18A···N10^iv^	0.93	2.50	3.346 (4)	151

Symmetry codes: (i) *x*+1, *y*+1, *z*; (ii) −*x*+1/2, *y*+1/2, −*z*+1/2; (iii) −*x*+3/2, *y*−1/2, −*z*+1/2; (iv) −*x*+1/2, *y*−1/2, −*z*+1/2.
